# Careful adjustment of Epo non-viral gene therapy for β-thalassemic anaemia treatment

**DOI:** 10.1186/1479-0556-6-10

**Published:** 2008-03-11

**Authors:** Emmanuelle E Fabre, Pascal Bigey, Yves Beuzard, Daniel Scherman, Emmanuel Payen

**Affiliations:** 1 Unité de Pharmacologie Chimique et Génétique, INSERM U640, Faculté de Pharmacie, 4 avenue de l'observatoire, 75006 Paris, France; 2 Unité de Pharmacologie Chimique et Génétique, CNRS UMR 8151, Faculté de Pharmacie, 4 avenue de l'observatoire, 75006 Paris, France; 3 Unité de Pharmacologie Chimique et Génétique, Université Paris Descartes, Faculté de Pharmacie, 4 avenue de l'observatoire, 75006 Paris, France; 4 Unité de Pharmacologie Chimique et Génétique, Ecole Nationale Supérieure de Chimie de Paris, 11 rue Pierre et Marie Curie, 75005 Paris, France; 5 Laboratoire de Thérapie Génique Hématopoïétique, Institut d'Hématologie (IUH), INSERM U733, Hôpital Saint-Louis, 75011 Paris, France

## Abstract

**Background:**

*In situ *production of a secreted therapeutic protein is one of the major gene therapy applications. Nevertheless, the plasmatic secretion peak of transgenic protein may be deleterious in many gene therapy applications including Epo gene therapy. Epo gene transfer appears to be a promising alternative to recombinant Epo therapy for severe anaemia treatment despite polycythemia was reached in many previous studies. Therefore, an accurate level of transgene expression is required for Epo application safety. The aim of this study was to adapt posology and administration schedule of a chosen therapeutic gene to avoid this potentially toxic plasmatic peak and maintain treatment efficiency. The therapeutic potential of repeated muscular electrotransfer of light Epo-plasmid doses was evaluated for anaemia treatment in β-thalassemic mice.

**Methods:**

Muscular electrotransfer of 1 μg, 1.5 μg, 2 μg 4 μg or 6 μg of Epo-plasmid was performed in β-thalassemic mice. Electrotransfer was repeated first after 3.5 or 5 weeks first as a initiating dose and then according to hematocrit evolution.

**Results:**

Muscular electrotransfer of the 1.5 μg Epo-plasmid dose repeated first after 5 weeks and then every 3 months was sufficient to restore a subnormal hematrocrit in β-thalassemic mice for more than 9 months.

**Conclusion:**

This strategy led to efficient, long-lasting and non-toxic treatment of β-thalassemic mouse anaemia avoiding the deleterious initial hematocrit peak and maintaining a normal hematocrit with small fluctuation amplitude. This repeat delivery protocol of light doses of therapeutic gene could be applied to a wide variety of candidate genes as it leads to therapeutic effect reiterations and increases safety by allowing careful therapeutic adjustments.

## Background

Therapeutic protein secretion by an *in vivo *transfected organ is one of the major gene therapy applications. One drawback to be avoided in such therapeutic strategy is the potentially deleterious secretion peak of therapeutic protein following DNA administration. The aim of this study was to adapt dosage and administration schedule of a chosen therapeutic gene to avoid this potentially toxic plasmatic peak.

Recombinant erythropoietin (rhEpo) injections are commonly used to treat anaemia linked to cancer treatment or chronic renal failure. However, rhEpo injections remain an expensive treatment which requires frequent delivery injection repeats and which can lead to anti-Epo antibodies production by the patient [1]. Therefore, erythropoietin (Epo) gene transfer appears to be a promising alternative for severe anaemia treatment since it requires less frequent treatment repeat and may allow sustained Epo secretion and constant patient coverage. Epo gene transfer has already been tested on normal animals and on anaemia animal models such as β-thalassemia and chronic renal failure models. To this end, various gene transfer strategies have been used such as *ex-vivo *strategies using engrafted transduced myoblasts or other cell types [2-4], viral strategies using adenovirus [5] adeno-associated virus [6,7], helper-dependent adenovirus [8], or non-viral strategies using naked DNA injection [9], poloxamer/DNA formulations [10] or naked DNA injection associated to electrotransfer [9,11-13]. In several of these studies, the gene dose transferred led to a maximum hematocrit value between 70 and 80% [6,9-13] which corresponds to potentially lethal polycythemia [6]. Therefore, in the particular case of Epo, an accurate level of transgene expression is required for safety reasons.

Temporal control systems of transgene expression have already been used in gene therapy preclinical experiments, including for Epo gene use [6,10,14,15]. These systems could avoid deleterious Epo secretion peak, but unsolved problems such as host immune response against the transactivator [10] or inducing agents adverse effects, are still restricting their use.

In order to avoid the toxic Epo plasmatic peak and to reduce plasmatic fluctuation amplitude, we decided to test different doses and administration schedules of an Epo encoding plasmid in anaemia treatment of β-thalassemic mice. Considering electrotransfer advantages in terms of safety, efficiency and cost, we chose this well-handled gene transfer method. Our previous experiment with β-thalassemic mice using intramuscular electrotransfer of an Epo encoding plasmid [9] led to a first estimation of transgene product kinetics and physiologic effects. Epo plasmatic level was found to reach a peak value within two weeks after gene therapy treatment and then to decrease approximately of 40%, 20% and 15% of this peak after 1, 2 and 3 months, respectively. This plasmatic Epo kinetics was roughly confirmed in normal mice by other studies with a secretion peak one week after electrotransfer [11,13]. However, Epo main physiologic effect on erythropoiesis which can be evaluated through hematocrit measurement remained intense for several months because of red blood cell half-life. Indeed, β-thalassemic mice hematocrit was still at the polycythemic value of 60% four months after 20 μg Epo-plasmid electrotransfer [9].

Considering those results, we have presently tested the therapeutic potential of repeated electrotransfer of suboptimal low Epo-plasmid doses in the β-thalassemic mouse model to restore and maintain a normal hematocrit without reaching toxicity.

## Methods

### Plasmid

The pCMV-Epo plasmid used for experiments was a pCOR plasmid [16] containing the mouse erythropoietin cDNA under the regulatory control of the hCMV E/P [17]. Plasmid large-scale production and double caesium chloride gradient ultracentrifugation used as purification method, were realised according to traditional molecular biology methods [18]. Plasmid construct was checked by restriction fragment length profile and sequencing.

### Animal experiments

Animal experiments were conducted following NIH recommendations. The β-thalassemic Hbb-thal1 mice [19] from the laboratory of Haematopoietic Gene Therapy (Saint Louis Hospital, Paris, France) were used for experiments. Two to four months female mice were separated into 6 groups: six Hbb-thal1 mice per group were used for the higher plasmid dose experiment, and eight Hbb-thal1 mice per group were used for the lower plasmid dose experiment. Mice were first anaesthetised by intra-peritoneal injection of 250 μl of a ketamine-xylazine solution (respectively 8.66 mg/ml and 0.31 mg/ml in 150 mM NaCl). Left rear legs were shaved and the Epo-plasmid solution was injected in the tibialis-cranialis muscle. The DNA solutions were diluted in 150 mM NaCl to contain the desired plasmid quantity in 30 μl: 1 μg, 1.5 μg, 2 μg, 4 μg and 6 μg, respectively, for the corresponding groups (meaning 50, 75, 100, 200 or 300 ng of plasmid per mouse gram, respectively). The DNA injection was immediately followed by application of eight electric pulses of 200 V/cm intensity, 20 ms duration and delivered at a frequency of 1 Hz, using plate electrodes and generator BTX ECM 830 (Genetronics™), as previously described [20].

### Sample collection, measurement and assay

Blood samples were collected by retro-orbital puncture of anaesthetised mice at desired time after plasmid electrotransfer. Hematocrits were measured using a standard micro-hematocrit method [21]. Mouse Epo assay was realised on serum samples using the EPO ELISA Medac^® ^kit (Medac™) based on cross-reaction with human Epo.

### Statistical analysis

Analysis of variance (ANOVA) and Fisher PLSD were used.

## Results and discussion

Our previous study of β-thalassemic mice demonstrated that electrotransfer of 1–10 μg Epo-plasmid doses were sufficient to induce a significant hematocrit increase. However, after a hematocrit burst depending on the dose of injected DNA during the first month after treatment, the hematocrit of treated mice started to decrease, and finally stabilised two months after electrotransfer. Surprisingly, this plateau was the same whatever the DNA dose used for gene transfer, and hematocrit still remained different from controls for at least 4 months [9]. Moreover, the 5 μg Epo-plasmid dose seemed to be the most appropriate since it led to normal hematocrit at peak value (approximately 45%). This hematocrit profile resulted from a shorter Epo plasmatic kinetics with peak of expression reached in less than 2 weeks and an expression level relative to this peak value of 40%, 20% and 15% respectively 1, 2 and 3 months after electrotransfer. Higher doses of Epo-plasmid led to hazardous unsafe hematocrit peak (60 to 80%). This study is then designed to slowly reach and maintain the hematocrit plateau and to avoid the initial hemarocrit burst.

**Figure 1:**
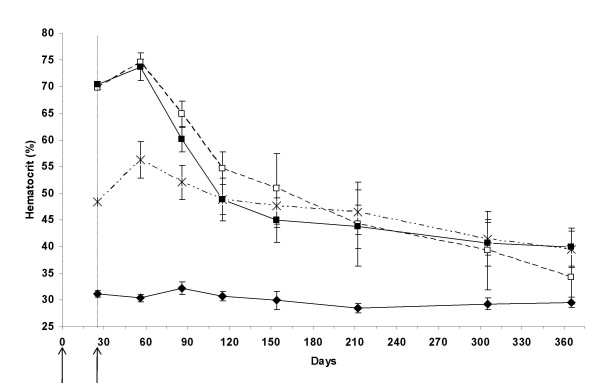
**Hematocrit of β-thalassemic mice electrotransfered twice with 2, 4 and 6 μg of Epo plasmid**. Hematocrit kinetics of β-thalassemic mice electrotransfered at day 0 and day 25 with 2 μg (cross), 4 μg (empty square) and 6 μg (solid square) Epo plasmid doses. The negative control (solid diamond) was realised by intramuscular injection of NaCl (150 mM) followed by electric pulse application. Error bars show standard error of mean (SEM). Arrows indicate electrotransfer applications.

To avoid a possible hematocrit busrt following the electrotransfer treatment, we decided to raise the hematocrit step by step by repetitive treatments with small doses of plasmid DNA. In our mind, the first treatment should be performed with a small dose of the plasmid that would be insufficient to reach a normal hematocrit value, but which should just raise it a little. The purpose of this first dose was to initiate the treatment. The following treatments would then performed to assess the possibility to raise the hematocrit a little bit more, closer to a normal value, and to maintain it to an almost constant value. To assess the DNA dose appropriate to this aim, we first evaluated Epo plasmid doses of 2, 4 and 6 μg per mouse which were electrotransfered at days 0 and 25 (fig 1). Maximum hematocrit values of 56.2% ± 3.2%, 74.5% ± 2.5% and 73.7% ± 2.4% respectively for the 2 μg, the 4 μg and the 6 μg groups, were reached two months after the first electrotransfer (fig 1). Therefore each dose led to polycythemia which was stronger for the 4 μg and 6 μg groups. Four months after the first electrotransfer, the hematocrit levels became equivalent between the three plasmid doses (no statistical difference), and kinetics showed similar slow decrease. Moreover, hematocrit level of each treated group remained significantly different from the control group up to 7.5 months (p < 0.05).

**Figure 2:**
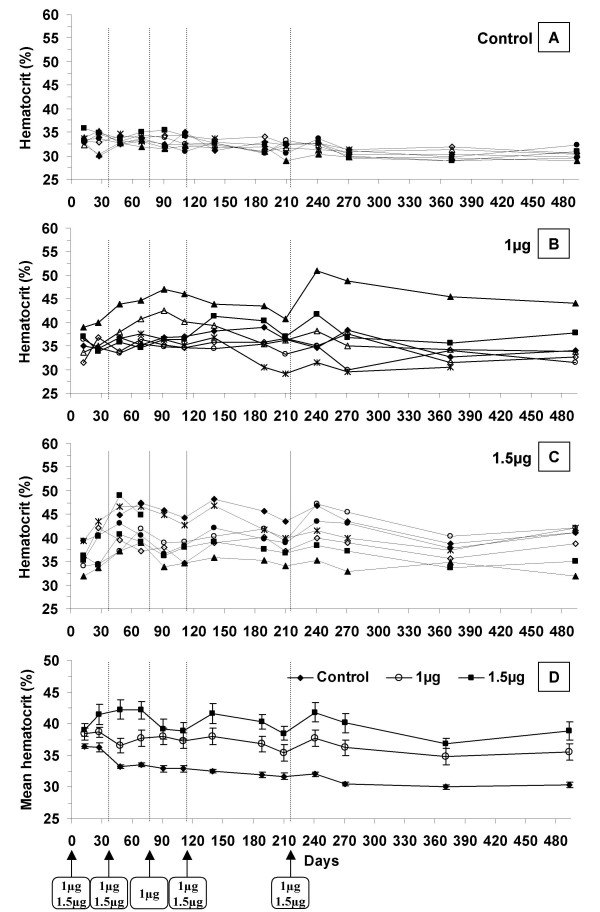
**Hematocrit of β-thalassemic mice after repeated muscular electrotransfer of 1 μg and 1.5 μg of Epo-plasmid**. Individual hematocrit kinetics of β-thalassemic mice electrotransfered with NaCl 150 mM solution for control group (2-A) or with 1 μg (2-B) and 1.5 μg (2-C) of Epo-plasmid for the other groups. Figure 2-D presents mean hematocrit of each group with standard error of the mean (SEM). Electrotransfer was performed at day 0, 34, 112 and 215 for the three groups. One additional electrotransfer was performed at day 77 for the 1 μg group. Arrows indicate electrotransfer applications.

Regarding those results, we decided to decrease plasmid doses down to 1 μg and 1.5 μg and to increase the time interval between electrotransfer treatments (fig 2). Electrotransfer of those plasmid doses was first repeated at day 34 and then according to hematocrit value. For additional treatments, we decided to use in each group the same dose used for the first treatment (i.e. 1 μg or 1.5 μg, respectively, for the two treated groups); treatments were performed when the mean hematocrit of the highest dose (1.5 μg) decreased around 40%. An additional treatment (day 80) was performed with the 1 μg group because we estimated that the hematocrit was too low. Following treatments were then performed at the same time points than for the 1.5 μg group.

**Figure 3:**
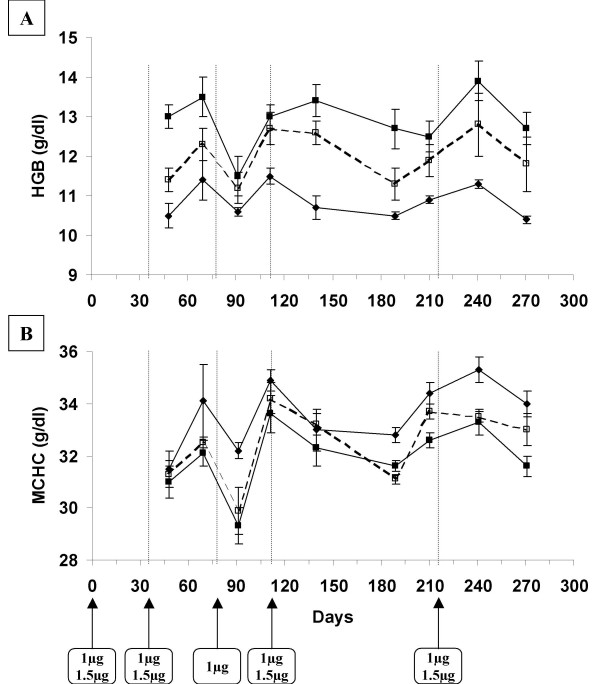
**Hemoglobin and MCHC evolutions after repeated muscular electrotransfer of 1 μg and 1.5 μg of Epo-plasmid**. Hemoglobin (HGB) evolution (2-A) and MCHC evolution (2-B) in β-thalassemic mice electrotransfered with NaCl 150 mM solution for control group (solid diamond) or with 1 μg (solid sphere) and 1.5 μg (solid square) Epo-plasmid doses for the other groups. Electrotransfer was performed at day 0, 34, 112 and 215 for the three groups. One additional electrotransfer was performed at day 77 for the 1 μg group. Error bars show SEM. Arrows indicate electrotransfer applications.

A hematocrit decrease of approximately 3% was observed in the control group between the beginning and the end of the experiment (fig 2-A) (p < 0.0001). As the study proceeded over 17 months, this is to be linked with anaemia escalation coming along with ageing in our β-thalassemic context, which as already been described [22]. The 1 μg dose delivered at day 0, 34, 77, 112 and day 215, led to significant hematocrit increase which was maintained between 35.4% and 38.7% during 10 months (fig 2-A and 2-B). The mean hematocrit value was significantly higher for this group than for the control group from day 69 (p < 0.05) to day 493 (p < 0.05). As compared to the β-thalassemic mice control group, the 1 μg administration schedule led to a progressive delta hematocrit increase during 3 months and then reached a 4–6% plateau value which was maintained until the end of the experiment. However, it appeared that with this dose we could not get any better than 39% (Fig 2). This dose is then definitely not sufficient for our goal to approach normal value. The administration schedule corresponding to 1.5 μg Epo-plasmid deliveries at day 0, 34, 112 and day 215 gave more promising results. An improved hematocrit value, between 38.4% and 42.3%, was steadily maintained for more than 9 months (fig 2-A and 2-C). The delta hematocrit, taking control group as reference, oscillated between 5.1% and 9.8% from one month after the beginning of the experiment to its end. Therefore, the hematocrit of the 1.5 μg group remained significantly higher than that of the control group from day 13 (p < 0.05) to day 493 at least (p < 0.001 at 17.6 months). Moreover, despite anaemia escalation coming along with ageing, similar hematocrit peak values were reached after the whole two firsts, the third and the fourth electrotransfers of the 1.5 μg Epo-plasmid dose. These hematocrit values were of 42.3%, 41.6% and 41.8%, and delta hematocrit values were of 9.0%, 9.0% and 9.8% respectively at days 48, 140 and 241 (no statistical difference). Therefore, the first two electrotransfers seemed to have an equivalent impact on hematocrit than the third and fourth treatments. mEPO plasmatic levels were measured, but no statistical difference could be highlighted between plasmatic Epo levels reached at days 48, 140 and 241 [additional file 1]. Actually, mEPO was detectable to levels close to the limit of detection of our ELISA kit. We believe this is not very surprising: as erythropoiesis is very sensitive to EPO levels, small changes in EPO levels may lead to very visible effects on hematocrit. As we targeted only small hematocrit increases, we did not expect high levels of circulating EPO. Instead, we believe that a statistically significant difference in hematocrit, which is the real physiological parameter we want to impact on, is much more relevant in this study. The other blood cell lineages were analysed from day 48 to day 271. According to time, significant increases in red blood cell count (data not shown) and hemoglobin concentration (fig 3-A) were observed. These increases were responsible for hematocrit increase. On the contrary, a decrease in mean corpuscular hemoglobin concentration (MCHC) was noticed when compared to the control at day 91 and then from day 189 to day 271 for the 1.5 μg group (p values of 0.002 on day 91, 0.005 on day 189, 0.002 on day 210, 0.01 on day 241 and 0.002 on day 271) and at day 91, 189 and 241 for the 1 μg group (p values of 0.02 on day 91, 0.001 on day 189 and 0.01 on day 241) (fig 3-B). Such a phenomenon has already been described in β-thalassemic mice treated with rhEpo [23] and might be related to iron deficiency [24]. The other lineage study did not reveal any variation (data not shown). In particular, we did not observe any variation in platelet counts, whereas it has already been found to be increased in patient with renal failure chronically treated with recombinant Epo [25].

This over one year study indicates that an appropriate administration schedule to treat β-thalassemic anaemia in mice could consist in a 1.5 μg Epo-plasmid dose electrotransfer firstly repeated after 5 weeks as an initiating dose to restore a normal hematocrit, and then repeated every 3 or 4 months to maintain this hematocrit level. The present experiment shows that repeated electrotransfer of low Epo-plasmid doses allows fine tuning of hematocrit response on a more than one year period. Looking at individual data, it appears that the hematocrit can be maintained at an almost constant level for each of the treated animal. This strategy allows to avoid the deleterious initial hematocrit peak and to maintain a normal hematocrit with small fluctuation amplitude. Furthermore, we may hypothesise that this administration schedule which leads to low Epo endogenous production, may limit humoral response which has been clearly correlated to transgene expression level [26]. Therefore, anti-Epo antibodies production coming along with host autoimmune reaction, which has already been described in non-human primate [7], might be avoided with the present repeated and light therapeutic protocol.

Regarding possible clinical applications of the electrotransfer technology, one may argue that repetitive use of electric pulses might be painful. As far as we know, no significant discomfort related to the electrotransfer technology in humans has been reported so far. Several clinical trials of electrochemotherapy were reported with a good tolerance to the electric pulses delivery. Electrochemotherapy has recently been evaluated in an European project (ESOPE) and validated for clinical use.

As far as muscle electrotransfer is concerned, at least two clinical trials have been approved and are being conducted in the area of cancer vaccination by two different companies, Ichor and Inovio (vaccination using tumor antigen). The results of these first in man studies should give us more details about the discomfort linked to this procedure.

## Conclusion

The present work indicates that plasmids can be delivered repetitively with little or none impairment of transgene delivery and expression, in opposite to viral vector mediated gene delivery. This repeated delivery protocol allows careful adjustments to reach the clinical endpoint and feedback for subsequent dose delivery. This safe treatment protocol could be applied to another anaemic context and extend to a wide variety of gene therapy applications using many candidate therapeutic genes such as growth factor genes.

## Competing interests

The author(s) declare that they have no competing interests.

## Authors' contributions

YB, DS, PB and EP carried out the design of the study. EEF and EP performed experimental protocols, assays and data collection. All the authors participated in data analysis. EEF drafted the manuscript with advices provided by PB. All the authors read and approved the manuscript.

## Supplementary Material

Additional file 1 Changes in erythropoietin (Epo) levels after repeated muscular electrotransfer of 1 μg and 1.5 μg of Epo-plasmid. the data provided shows the mean EPO level reached in mice following the electrotransfer treatments, for all three groups of mice (ie, control group, 1 μg treated group and 1.5 μg treated group). Mouse Epo changes in β-thalassemic mice electrotransfered with NaCl 150 mM solution for control group (solid diamond) or with 1 μg (solid sphere) and 1.5 μg (solid square) Epo-plasmid doses for the other groups. Electrotransfer was performed at day 0, 34, 112 and 215 for the three groups. One additional electrotransfer was performed at day 77 for the 1 μg group. Arrows indicate electrotransfer applications. The EPO ELISA Medac^™ ^kit was used to measure mouse Epo based on cross-reaction (detection limit of 25 mU/ml for human Epo). Data are presented as mean Epo levels with standard error of the mean (SEM). Click here for file
