# Supplementary figures and images for: Careful adjustment of Epo non-viral gene therapy for β-thalassemic anaemia treatment
Source: Genet Vaccines Ther. 2008 Mar 11;6:10. doi: 10.1186/1479-0556-6-10 (PMC2276190; doi:10.1186/1479-0556-6-10)

## Slide 1
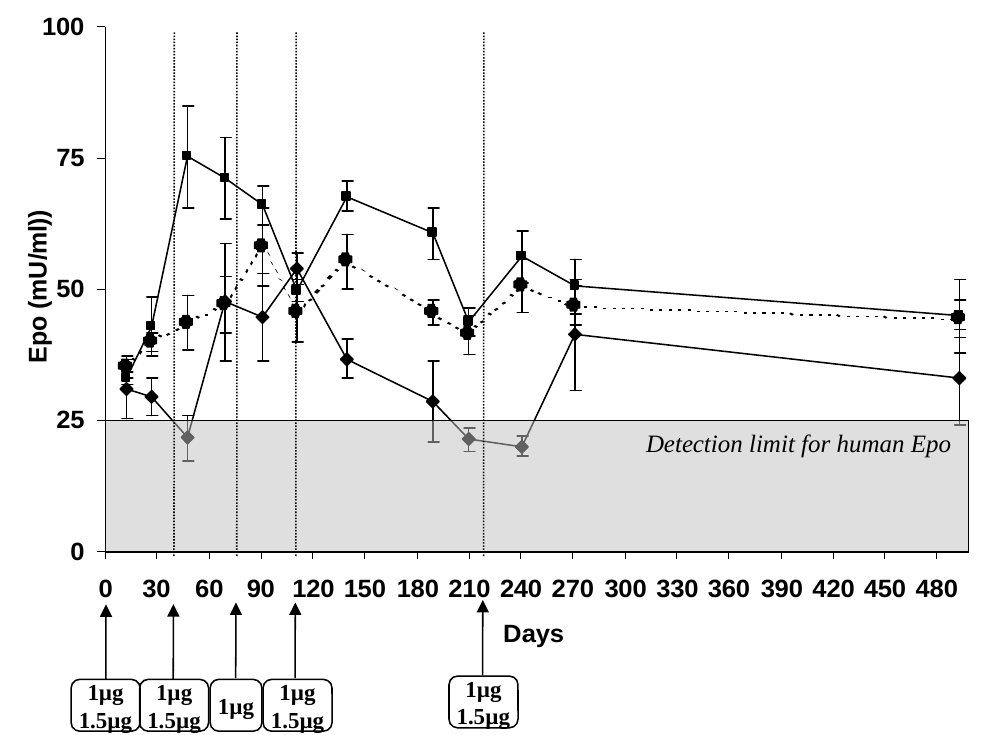

1µg
1.5µg
1µg
1.5µg
1µg
1.5µg
1µg
1µg
1.5µg
Detection limit for human Epo

Supplement: Additional file 1 — Changes in erythropoietin (Epo) levels after repeated muscular electrotransfer of 1 μg and 1.5 μg of Epo-plasmid. the data provided shows the mean EPO level reached in mice following the electrotransfer treatments, for all three groups of mice (ie, control group, 1 μg treated group and 1.5 μg treated group). Mouse Epo changes in β-thalassemic mice electrotransfered with NaCl 150 mM solution for control group (solid diamond) or with 1 μg (solid sphere) and 1.5 μg (solid square) Epo-plasmid doses for the other groups. Electrotransfer was performed at day 0, 34, 112 and 215 for the three groups. One additional electrotransfer was performed at day 77 for the 1 μg group. Arrows indicate electrotransfer applications. The EPO ELISA Medac™ kit was used to measure mouse Epo based on cross-reaction (detection limit of 25 mU/ml for human Epo). Data are presented as mean Epo levels with standard error of the mean (SEM). [file 1479-0556-6-10-S1.ppt]
